# Optimized Laboratory Maintenance and Functional Testing of *Chara braunii*

**DOI:** 10.1002/cpz1.70279

**Published:** 2025-12-30

**Authors:** Katarina Kurtović, Jan Petrášek, Stanislav Vosolsobě

**Affiliations:** ^1^ Department of Experimental Plant Biology, Faculty of Science Charles University Prague Czech Republic; ^2^ Institute of Experimental Botany Czech Academy of Sciences Prague Czech Republic

**Keywords:** *Chara braunii*, cultivation, germination, in vivo staining, propagation, streptophyte algae, Raspberry PI

## Abstract

Chara braunii is an emerging model species for studying plant evolution and development. Various members of the Chara genus have been used for pioneering studies of electrophysiology, cytoplasmic streaming, and cell biology. However, many studies have been limited by challenges, such as overgrowth with epiphytes or non‐continuous growth throughout the year. Here, we present protocols for the cultivation, germination, and in vivo staining of C. braunii NIES‐1604 to overcome these limitations. We constructed the custom‐made cultivation chamber equipped with the illumination unit that allows for the precise simulation of natural conditions and continuous cultivation of C. braunii NIES‐1604 throughout the year. By determining the optimal stratification period for C. braunii NIES‐1604 oospores, we achieved a germination rate of 26%. Further, we present fluorescence‐based in vivo staining protocols for routine in vivo staining of cellular structures including the plasma membrane, cell wall, and nuclei. Altogether, our affordable method of controlled cultivation of C. braunii NIES‐1604 and other procedures serve as a valuable resource for establishing Chara cultures in laboratory settings and advancing its use as a model organism. © 2025 The Author(s). *Current Protocols* published by Wiley Periodicals LLC.

**Basic Protocol 1**: Construction of a cultivation chamber for *C. braunii* NIES‐1604 growth

**Basic Protocol 2**: Vegetative propagation of *C. braunii* NIES‐1604

**Basic Protocol 3**: Germination of *C. braunii* NIES‐1604 oospores

**Basic Protocol 4**: In vivo staining of *C. braunii* NIES‐1604 with fluorescent dyes

## INTRODUCTION

Understanding how land plants (embryophytes) evolved from aquatic ancestors requires experimental systems that represent their closest algal relatives, the streptophyte algae (Bowman, 2022; Delwiche & Cooper, 2015) Within this group, *Chara braunii* stands out as a particularly valuable model. Although not the closest relative to land plants, its phylogenetic position makes it informative for studying traits shared with embryophytes, while its large, structurally specialized cells enable unique insights into plant cell biology (Beilby et al., 2022; Nishiyama et al., 2018). However, to fully harness this potential, reproducible and optimized protocols for cultivation, germination, and cellular imaging are essential (Domozych & Bagdan, 2022; Holzhausen et al., 2022).

*Chara*’s distinctive giant internodal cells have long served as a valuable model in plant physiology research (Beilby et al., 2022). With diameters up to 1 mm and several centimeters in length, these cells offer a unique system for investigating fundamental cellular processes, including cytoplasmic streaming, membrane transport, and cell expansion (Domozych et al., 2016). The availability of genomic resources for *C. braunii* strain S276 (Nishiyama et al., 2018) has fueled transcriptomic studies (Heß, Heise, et al., 2023; Heß, Holzhausen, et al., 2023), and a cultivation protocol (Holzhausen et al., 2022).

Despite these advances, current methods face limitations. Germination and cultivation protocols developed for one *Chara* species or a strain are often not transferable to others (Kurtović et al., 2024). Dormancy‐breaking requirements vary widely, from prolonged cold stratification (e.g., *Chara hispida* and *Chara globularis*) (Forsberg, 1965) to immediate germination after harvest (*Chara contraria*) (Sabbatini et al., 1987). Furthermore, *Chara* cell biology trails that of land plants (Domozych & Bagdan, 2022). While charasomes, i.e., specialized, convoluted plasma membrane domains of internodal cells, are well described (Absolonova et al., 2019; Chau et al., 1994; Klima & Foissner, 2008; Schmölzer et al., 2011) plasma membrane of other cell types, e.g., rhizoids and antheridia, remain poorly characterized.

Here, we present optimized protocols for *C. braunii* strain NIES‐1604, a unialgal strain publicly available from the NIES collection and supported by existing RNA‐seq data (GSE275253). We developed a custom cultivation chamber to enable continuous growth under controlled laboratory conditions and established germination parameters, identifying a minimal cold stratification period of 2 months and an optimal period of 6 months. We further demonstrate live‐cell staining approaches for visualizing diverse cellular structures in *Chara*, providing tools to study its cellular architecture and physiology *in vivo*.

Compared with previously published protocols for *C. braunii* cultivation (e.g., Holzhausen et al., 2022), the procedures described here introduce several improvements. Our custom‐built cultivation chamber enables continuous year‐round growth under reproducible laboratory conditions, overcoming the seasonality that often limits *C. braunii* maintenance. In addition, the protocols emphasize cost‐effective and easily accessible materials, allowing for broad adoption across laboratories. By integrating optimized germination, vegetative propagation, and in vivo staining methods into a single workflow, we provide a standardized framework that improves reproducibility and comparability between studies. These protocols generate both qualitative (microscopy‐based structural characterization) and quantitative (germination rates under different stratification regimes) data. Compared with existing methods, our approach combines cultivation, germination, and live‐cell imaging in a single workflow, reducing variability and enabling reproducibility across laboratories. The primary advantage is the accessibility of the NIES‐1604 strain and the adaptability of the protocol to controlled environmental setups while the main limitation is that cold stratification requirements may not generalize to all *Chara* species.

The methods below describe four protocols. Basic Protocol 1: Construction of cultivation chamber for *C. braunii* growth, describes the design, assembly, and environmental parameters for maintaining *C. braunii* cultures under controlled laboratory conditions. Basic Protocol 2: Vegetative propagation of *C. braunii*, outlines methods for maintaining and expanding cultures through controlled fragmentation and regrowth. Basic Protocol 3: Germination of oospores, details optimal and minimal cold stratification periods to break dormancy and maximize germination rates. Basic Protocol 4: In vivo staining of *C. braunii* with fluorescent dyes, provides step‐by‐step instructions for labeling specific cellular structures for live‐cell imaging. Together, these methods establish a reproducible workflow for cultivating, propagating, germinating, and visualizing *Chara* in laboratory settings.

## Basic Protocol 1 CONSTRUCTION OF A CULTIVATION CHAMBER FOR C. braunii GROWTH

Cultivation of *C. braunii* benefits from stable and reproducible environmental conditions, particularly with respect to light intensity, temperature, and photoperiod. For this purpose, a dedicated cultivation chamber equipped with programmable LED illumination and controlled air circulation is recommended, as it provides the most uniform growth conditions. However, *Chara* can also be maintained under standard aquarium lights, though growth may be less consistent. Below, we provide a detailed protocol for constructing the cultivation unit, including assembly of the illumination system, wiring of electronic components, and set up of the control software.

### Materials

Cultivation box, e.g., a refrigerator with temperature control up to 25°C
LED strips of different colors (see Table 1)
2‐wire and 4‐wire cables for single and RGB LED strips, respectively
Heat shrink tubing, 10‐mm diameter
Aluminum LED profiles for all LED strips or an aluminum plate of appropriate dimensions for all strip mounting
Double‐face mounting tape for attaching LED profiles to the cultivation box
12 V power supply for LED strips with sufficient power (100 W for setup in Table 1)
*Any ATX power supply from an old computer can be used. It also allows connecting the Raspberry Pi to its 5.0 V output*.
Six IRF520 MOSFET driver module (EAN, 5903351241212)
*Six individual modules can be replaced for example by one 8‐channel MOSFET module; support of 3.3 V input voltage is necessary in all cases*.
Raspberry PI minicomputer (e.g., EAN, 4250236816296)
*Any Arduino clones can be used. The advantages of Raspberry PI are the easier programming thanks to its own operating system, easy connection to screen, keyboard, mouse and network, and presence of large internal SD storage*.
DSI LCD touch display for Raspberry PI (EAN, 614961956940)
*A display is optional. This screen can be attached directly to the Raspberry PI and together at the surface of the cultivation box. This enables to show the actual status of the cultivation box and easy setup. Alternatively, Raspberry PI can be connected to any HDMI‐equipped screen*.
Set of DuPont cables (Female‐Female) for connecting the Raspberry PI with MOSFET modules (e.g., EAN, 5063200231570)
DuPont terminal block pin headers for multiplying the +3.3 VCC output of Raspberry Pi to all MOSFET modules (e.g., AliExpress, 1005006998044499)
*A cable splitter can be easily prepared by soldering twisted DuPont cables or using a small piece of stripboard*.
Nylon standoff kit and plastic box for enclosing the Raspberry PI and MOSFET modules.
*A plastic food storage box with sufficient openings for ventilation can be used*.
PC fan (e.g., EAN, 4710614532779)
*To equalize the temperature inside the box and avoid excessive heating under the LED strips, it is a good idea to mount a 12 V PC fan in the bottom part of the box*.
USB flash drive

**Table 1:** Excitation and Emission Spectra of LED Strips Used in the Custom Cultivation Box

LED strip	Power (W/m)	No. of LEDs (m^−1^)	Emission *λ* (nm)	No. of LEDs (used for 800 cm^2^)	Relative intensity (sun‐like setup)
RGB SMD*^a^*	14.4	60	425‐490 (B) 485‐565 (G) 600‐645 (R)	36*^a^*	0 100 0
Day white 92 CRI	20	120	435‐480*^b^* 480‐740	108	60
UV	14.4	60	385‐420	18	90
Plant growth	12	60	430‐470*^b^* 580‐770	36	20

^*a*^ RGB strip contains combined LEDs emitting three different colors.

^*b*^ LEDs have two peaks of emission.

#### Construction of the unit

1 Drill openings in the rear panel of the cultivation box to accommodate cable routing for the LED strips or implement an alternative cable management solution.
2 Cut the LED strips to the desired length and solder them to appropriately sized 2‐ or 4‐wire cables. Insulate the connections using heat shrink tubing.
3 Attach the LED strips to the pre‐cut LED profiles using their adhesive backing. Then, secure the profiles to the upper side of the cultivation box using short pieces of mounting tape.
4 Drill openings in the selected electronics box for the LED strip cables, the 5 V power supply for the Raspberry Pi, and the 12 V power input.
5 Fix the MOSFET modules to the bottom of the plastic box using a standoff kit. Mount the Raspberry Pi to the display and then mount this assembly into the opening in the lid of the plastic box using screws.
6 Connect the GND pin of the six MOSFET modules with one of the GND pinouts of Raspberry Pi (Pins 6, 9, 14, 20, 25, 30, 34, or 39) using DuPont cables.
7 Connect the VCC pins of the six MOSFET modules with the 3.3 V pinouts of Raspberry Pi (Pins 1 and 17) using the terminal block header or cable splitter.
8 Connect the SIG pins of the six MOSFET modules with one of the GPIO pinouts of Raspberry Pi (e. g. Pins 7, 11, 12, 13, 15, and 16 for GPIOs 4, 17, 18, 27, 22, and 23, respectively).
9 Interconnect the VIN inputs of the MOSFET module and connect one of them to the +12 V output of the LED power supply. Do the same with the GND inputs, connecting one of them to the –12 V (GND) terminal of the power supply.
10 Connect the cables from the LED strips to the + and – outputs of the MOSFET modules. In the case of an RGB strip, there are individual – inputs for each color and common + output. Connect the + cable to the + outputs of one MOSFET module and link the + outputs of the other two MOSFET modules to it.
11 Connect the 5 V power supply to the Raspberry Pi.
12 Connect the fan to a 12 V power supply and fix it inside the box, e.g., using a hot glue gun.

#### Programming of the Raspberry PI

13 Full Python code for the illumination unit is deposited in the repository at: https://github.com/vosolsob/illumination.
14 Download the illumination.py file to a portable device (USB flash drive).
15 Connect a mouse, keyboard, and the USB flash drive to the Raspberry Pi.
16 Follow the instructions in the repository.
17 For *C. braunii*, set the temperature regime to 19° and 22°C for the dark and light periods, respectively.

## Basic Protocol 2 VEGETATIVE PROPAGATION OF C. braunii NIES‐1604

Vegetative propagation of *C. braunii* requires stable cultivation conditions to ensure reliable regeneration and growth. While the use of a dedicated cultivation chamber with programmable illumination and controlled temperature is recommended for achieving uniform and reproducible results, *Chara* can also be maintained under standard aquarium lights when such a system is not available. The following protocol for *C. braunii* NIES‐1604 is a modified version of the Characeae protocol (SWCN‐4). The strain, obtained from the National Institute for Environmental Studies (NIES) in Tsukuba, Japan, was selected for its unialgal nature. Propagation in culture tubes is particularly useful for experimental treatments or when individual thalli are required. The steps below describe preparation of the soil–sand medium, sterilization of water and tools, handling of thallus explants under sterile conditions, and maintenance of cultures over time.

### Materials

Garden soil
H_2_O, sterile deionized
Silica sand
*C. braunii* adult thalli, 2‐ to 3‐weeks old
70% ethanol

26 × 180–mm glass tubes with round bottom
500‐ml blue‐cap bottles
Aluminum foil
Autoclave
30‐ and 23‐cm tweezers (Duchefa, F3003 and F300)
Long scissors, 25‐cm (e.g., Metzenbaum‐Nelson)
Scalpel
10‐cm glass Petri dishes, 8 to 10
30‐ml sterile syringe (Braun Omnifix Luer lock)
100‐ml Erlenmeyer flask
Laminar flow hood

#### Preparation of the medium

1 Add 1 ml of garden soil to a glass tube and dampen it slightly with a few drops of deionized water. On top, add 4 to 5 ml of silica sand that has been washed with deionized water and drained.
  For larger quantities, cultures, Chara can be grown in 250‐ml blue‐cap bottles using the same medium as for tubes.
2 Create a cap from aluminum foil and cover the tubes. Prepare 10 to 15 tubes.

#### Sterilization

3 Sterilize the soil by autoclaving it twice at 121°C for 30 min.
  Soil can be sterilized in two rounds with overnight cooling in between.
4 Wrap Petri dishes, tweezers, scissors, and a scalpel in aluminum foil. Sterilize these items in the autoclave for 30 min at 121°C or program for “wrapped material”.
5 Sterilize two 500‐ml bottles of deionized water.

#### Filling the tubes with deionized water

6 After the second sterilization, transfer the soil‐filled tubes, sterile deionized water, and a sterile syringe to a sterile bench disinfected with UV light or 70% ethanol. Use one bottle of sterile water for filling the tubes and the other for propagation.
7 Fill the tubes with sterile deionized water. To avoid disturbing the soil and to keep the medium clear, do not pour water directly from the bottle. Instead, transfer sterile water to a sterile Erlenmeyer flask and use a sterile 30‐ml syringe to fill each tube slowly with 25 to 35 ml water.
  It is important to sterilize soil and water separately to avoid formation of dense soil extract in medium, which is inhibitory for thalli growth.
8 Proceed immediately with propagation. Perform the following steps in a laminar flow hood.
  Prepare and sterilize medium and water just before use to reduce contamination; older medium contaminates more easily.

#### Propagation

9 Unwrap Petri dishes and tweezers and place them in one corner of the sterile bench. If tweezers touch a non‐sterile surface, briefly flame them.
10 Place a Petri dish in the center of the bench and pour some sterile water into it. Bring a 2‐ to 3‐week‐old *C. braunii* culture in tubes to the bench. Surface sterilize the tubes using 70% ethanol, allow ethanol to evaporate, then remove the aluminum cap and briefly flame the tube opening.
11 Cut *Chara* thalli from its medium with sterile scissors, collect the cut segments with a tweezer, and transfer to the Petri dish with sterile water.
12 Rinse *Chara* in the same Petri dish 2 to 3 times with sterile water to reduce surface contamination. If soil particles are present, transfer the thalli to a clean Petri dish with water.
13 Keep *Chara* submerged to avoid drying of the thallus. Cut the thallus into 2 to 3 node explants with a scalpel or scissors.
  Chara regeneration varies along the thallus: the middle section regenerates fastest, followed by the tip, while the base is least effective. Explants with a thallus tip resume growth quickly, while decapitated explants regenerate a new thallus before elongation.
14 Using long tweezers, carefully place 1 to 2 *Chara* explants per tube with prepared medium. Hold the thali with tweezers by the lowest cut internode. Ensure the bottom node is buried under the sand, as contact with soil is essential for rhizoid formation.
15 Seal tubes with aluminum caps, leaving them slightly loose for airflow.
  Air bubbling is unnecessary and may hinder Chara growth.
16 Sterilize tweezers between each tube by briefly passing them over a flame.
17 Place propagated *Chara* at 19° to 22°C, 14 hr light/10 hr dark cycle, 30 µmol m^−2^ s^−1^ (for the protocol for the custom‐made cultivation chamber).

#### Changing the water

18 Change the water for adult *Chara* every 2 to 3 weeks for optimal growth.
19 Follow the same procedure as described in the “Filling the tubes with deionized water” section above.
20 Carefully pour out the old water and refill with fresh sterile water using a sterile syringe.
21 In the second replacement of water, increase its volume to 40 to 50 ml because of thali elongation (the maximum capacity is ∼60 ml).
  More frequent water change promotes Chara growth.

## Basic Protocol 3 GERMINATION OF C. braunii NIES‐1604 OOSPORES

Germination of *C. braunii* oospores provides an entry point for establishing new cultures and enables experiments starting from the sexual stage of the life cycle. Consistent cultivation conditions, ideally within a dedicated chamber with controlled light and temperature, support more uniform germination, although oospores can also germinate under aquarium lighting. This procedure describes harvesting and stratification of mature oospores, their disinfection prior to use, and sowing either on soil/sand medium or in agar plates. While germination in agar plates typically begins within 2 weeks, full thalli development requires transfer to a nutrient‐containing soil medium and may take 1 month.

### Materials

4‐ to 5‐week‐old mature thalli with oospores
H_2_O, sterile deionized
Tween‐20
Commercial bleach (typically 5% NaOCl)
Soil/sand medium
0.5% agar medium for germination (see recipe)

10‐cm glass Petri dishes, 8 to 10
Gloves
1.5‐ml microcentrifuge tube
Refrigerator
Tweezers, fine‐tipped (e.g., Dumont #5)
Laminar flow hood
Pipettes and sterile pipette tips
60‐mm sterile plastic Petri dishes

#### Harvest

1 Harvest 4‐ to 5‐week‐old mature thalli along with oospores and place the material in a closed glass Petri dish at room temperature to desiccate for 2 to 3 weeks. The biomass will dry out and adhere to the Petri dish.
  Mature oospores are dark brown/black, and thalli should be yellowing and no longer elongating.
2 Using gloves, gently rub the dried biomass in the Petri dish to partially detach the oospores from the thalli
3 Transfer the oospores into a 1.5‐ml microcentrifuge tube and add deionized water to rehydrate them.
4 Place the oospores in deionized water in the refrigerator at 4°C for 6 months to break dormancy.
  Stratified oospores are not sterile, but this did not affect germination in our conditions. Prior to oospore disinfection it is possible to remove excess biomass under a binocular microscope, manually, using micro tweezers. Handle gently, as hydrated oospores are fragile.

#### Disinfection

5 Perform the following steps in a laminar flow hood. Briefly wash the stratified oospores in a 1.5‐microcentrifuge tube with 0.1% Tween‐20 in sterile deionized water.
6 Wait a ~5 seconds for the oospores to sediment at the bottom of the tube. Then, carefully remove the detergent by pipetting (using sterile 1‐ml tips) and replace it with sterile deionized water. Repeat this procedure at least 10 times.
7 Continue the disinfection process by adding diluted commercial bleach (1:10 in water) and incubating for 10 min.
8 Wash the oospores with sterile deionized water 5 to 6 times.
9 Store the disinfected oospores in sterile deionized water at 4°C if they are not going to be sown immediately.

#### Germination medium and sowing

10 Sow the oospores on the surface of a soil/sand medium with deionized water (see Basic Protocol 2 for vegetative propagation) using a 1‐ml pipette with a cut tip or proceed to step 11.
11 Alternatively, sow the oospores in autoclaved 0.5% agar medium in sterile 60‐mm sterile plastic Petri dishes ensuring that the oospores are embedded in the agar to prevent drying out.
  Germination will begin ∼14 days after sowing. In agar medium oospores will germinate but they will not develop further than the protonema stage due to lack of nutrients; upon germination it is required to transfer them to soil medium to induce thallus development.

## Basic Protocol 4 IN VIVO STAINING OF C. braunii NIES‐1604 WITH FLUORESCENT DYES

Fluorescent staining of *C. braunii* thallus enables visualization of cellular structures and developmental processes using confocal microscopy. Depending on the experimental aim, different developmental stages, e.g., thallus tips, side branches, germinated oospores, or generative organs, can be prepared for staining. The following protocol for *C. braunii* NIES‐1604 describes preparation of plant material, incubation with common fluorescent dyes (FM 1‐43, FM 4‐64, Calcofluor White, and Hoechst 33342), and subsequent imaging. Attention to sample handling is essential, as *Chara* explants are fragile and staining volumes can be adjusted according to explant size and experimental requirements. This approach provides a reliable method for studying cell structure and organogenesis in *Chara*.

### Materials

2‐ to 3‐week‐old *C. braunii* cultures or 2‐week‐old germinated oospores
H_2_O, deionized
Fluorescent dyes (see recipe)
Phosphate‐buffered saline (PBS) (see recipe)

Long scissors
Long tweezers (Duchefa, F300)
40‐mm glass Petri dish
Binocular microscope
Fine‐tipped tweezers (e.g., Dumont #5)
Timer
Microscopic slide
Cover slips
Confocal or fluorescent microscope with camera

#### Material preparation (for different types of samples)

1 For thallus tip staining, cut the thallus tips of *Chara* using long scissors or tweezers. For observation of rhizoids, carefully remove the entire thallus from the substrate with long tweezers to preserve the rhizoids. Place the material in a 40‐mm Petri dish containing sterile deionized water and gently wash the thalli to remove surface contamination or sand particles. Adjust the size of the Petri dish if working with larger thalli.
  Due to the relatively high price of FM dyes, the staining volume can be reduced by using a smaller Chara explant and a smaller volume of a staining container. Due to the fragility of the tissue, we recommend using flat containers, e.g., microcentrifuge tubes. In addition, the dye concentration can be reduced if needed to achieve a less intense fluorescence signal.
2 For study of side branch development, cut the thallus tip and leave the decapitated thallus in its soil/sand culture medium for at least 1 week to initiate and form side branches.
  Alternatively, use propagated decapitated segments that are 2 weeks post–propagation. Remove the segments completely from the medium using long tweezers. Retain only the bottom node (previously under the sand surface) and cut away the remaining thallus. Use the entire node for staining (it should contain several side branches). Proceed with staining as with thallus segments.
3 For germinated oospore staining, excise the oospore germinated in agar as an agar block. Under a binocular microscope, use fine‐tipped tweezers to carefully remove the germinated oospores from the agar and place them in a Petri dish containing sterile deionized water.
4 For generative organ staining, leave the *Chara* thallus in its culture medium for 1 to 3 weeks. Cut the thallus from the medium, ensuring the branchlets are intact. The branchlets will contain antheridia and oogonia in various stages of development, with the bottom branchlets containing more mature antheridia and the apical part containing the least mature. Briefly wash the material in sterile deionized water.

#### Staining

5 Transfer the material from sterile water to a 40‐mm Petri dish containing incubation solution with fluorescent dye and start timer.
6 Incubate the samples with FM 1‐43, FM 4‐64, and calcofluor for 2 to 5 min, and with Hoechst 33342 for 5 to 10 min. Carefully replace the incubation solution with PBS or water 2 times as a washing step.
7 Immediately proceed with microscopic imaging.

#### Confocal or fluorescence microscopy

8 Place the stained material on a microscopic slide with a drop of water. Add a coverslip of an appropriate size.
9 For oospores, place a coverslip at both ends of a microscopic slide as a spacer and then another cover glass on the top.
  Oospores might burst if excessive pressure is applied with the coverslip.
10 Perform confocal imaging using a Leica SP8 laser scanning confocal microscope equipped with a HC PL APO 63×/1.20 water‐immersion objective and a HyD detector.
  Imaging can be performed on any other confocal microscope.
11 Typical laser power was kept <2% of maximum output, with pinhole set to 1 Airy unit, line averaging of 2 to 3. For fluorescence microscopy, exposure times were 200 to 400 ms, adjusted to avoid pixel saturation.
12 Excitation and emission spectra for FM 1‐43, FM 4‐64, calcofluor white, and Hoechst 33342 are listed in Table 2.

**Table 2:** Excitation and Emission Spectra of Fluorescent Dyes Used for Staining *C. braunii* NIES‐1604

Fluorescent dye	Target structure	Excitation (nm)	Emission (nm)	Recommendations
FM 1‐43	Plasma membrane	488	500‐550 570‐620*^a^*	High signal‐to‐noise ratio, minimal chloroplast autofluorescence; recommended for photosynthetic tissue; non‐compatible with yellow and red fluorescence emitting dyes
FM 4‐64	Plasma membrane	561*^b^* 514	570‐620*^c^*	Lower signal‐to‐noise in green tissues due to chlorophyll background; suitable for rhizoids and protonema
Calcofluor White	Cell wall	405	420‐480	Bright signal; can be combined with FM dyes; avoid over‐exposure to prevent photobleaching
Hoechst 33342	Nuclei	405	420‐480	Same settings as Calcofluor White; shorter exposure (≤200 ms) recommended to minimize background

^*a*^ Emission in 570‐620 nm band is stronger.

^*b*^ Excitation by 561 nm is significantly more effective.

^*c*^ A red emission band interferes with autofluorescence of chloroplast (beginning sharply at 640 nm).

## REAGENTS AND SOLUTIONS

### Agar medium for germination, 0.5%

0.5 g plant agar (Duchefa, P1001)
100 ml deionized H_2_O
Mix slightly and autoclave 30 min at 121°C
Prepare fresh or store up to 3 months at room temperature

### Fluorescent dyes

*FM 1‐43, 20 mM*
1 mg lyophilized FM 1‐43 powder (Thermo Fisher, T3163)
80 µl dimethyl sulfoxide (DMSO) or deionized H_2_O
Create 10‐µl aliquots and store up to 5 months at –20°C in the dark until ready to use
*FM 4‐64, 20 mM*
1 mg lyophilized FM 4‐64 powder (Thermo Fisher, T3166)
80 µl dimethyl sulfoxide (DMSO) or deionized H_2_O
Create 10‐µl aliquots and store up to 5 months at –20°C in the dark until ready to use
*Calcofluor White, 1 mg/ml*
1 mg Calcofluor White (PhytoTech Lab, C1933)
1 ml deionized H_2_O
1 drop of 1 N NaOH
Create 10‐µl aliquots and store up to 5 months at –20°C in the dark until ready to use
*Hoechst 33342, 1 mg/ml*
10 mg Hoechst 33342 (Invitrogen, H1399)
10 ml deonized H_2_O
Create 10‐µl aliquots and store up to 5 months at –20°C in the dark until ready to use
*Incubation solutions*

Prepare 5 ml of incubation solution in a 15‐ml conical tube (e.g., TPP, 91015). For FM 1‐43 or FM 4‐64 prepare 2 µM in PBS (1:10,000; see recipe). For Calcofluor White and Hoechst 33342 prepare 1 µg/ml solution in PBS (1:1000; see recipe). Pour the incubation solution into a 40‐mm Petri dish. Always prepare incubation solutions fresh prior to the staining. The dyes are light sensitive therefore, during the experiment, keep the incubation solution covered from the direct light by wrapping them in aluminum foil.

### Phosphate‐buffered saline (PBS), pH 6.9

0.818 g NaCl (140 mM final; Current Protocols, 2006)
0.022 g KCl (2.95 mM final; Current Protocols, 2006)
0.0324 g KH_2_PO_4_ (2.38 mM final; Current Protocols, 2006)
0.108 g Na_2_HPO_4_· (7.61 mM final; Current Protocols, 2006)
100 ml deonized H_2_O
Store up to 6 months at 4°C

## COMMENTARY

### Background Information

**Figure 1:**
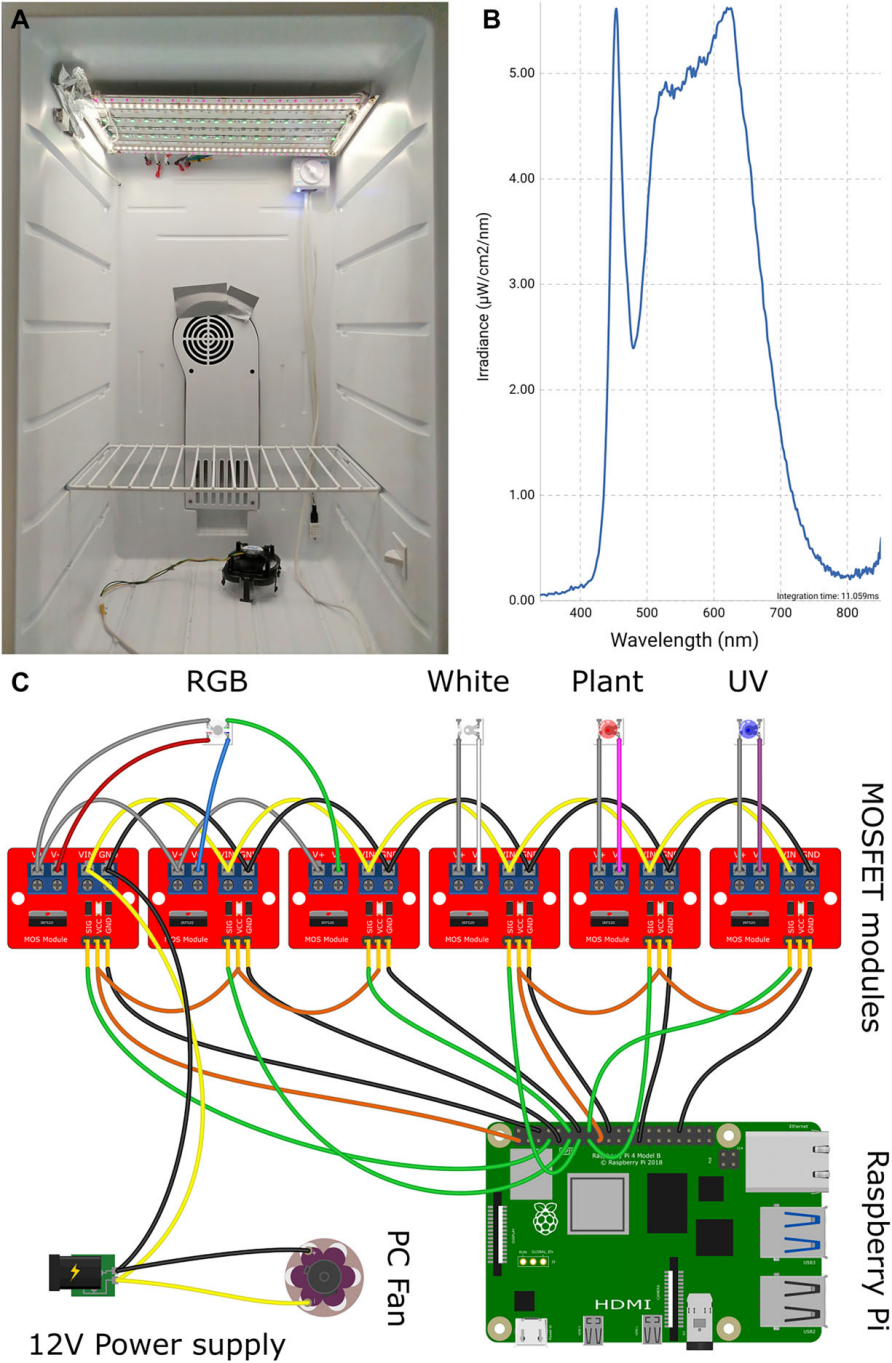
Cultivation chamber designed for continuous growth of *C. braunii*. (**A**) A custom‐made cultivation chamber built from a refrigerator. (**B**) Light spectrum of the cultivation chamber measured with SpectraPen mini (Photon Systems Instruments). (**C**) A wiring diagram of a custom illumination unit for *C. braunii* cultivation. GND lines are black (or colored in case of LED connections), 12 V lines are blue (or grey), 3.3 V lines are orange, and signal lines are green.

Our custom‐based cultivation chamber allows the culture of *C. braunii* consistently throughout the whole year. The protocol is specifically aimed at growing *C. braunii* in test tubes, which is suitable for experimental setups where individual plants are needed, such as growth experiments with different treatments. Exposing *C. braunii* NIES‐1604 to conditions used for a cultivation of *Arabidopsis thaliana* led to inhibition of its growth while maintaining under natural sunlight at the south‐oriented window resulting in the rapid growth of *Chara*. To standardize the cultivation conditions, we constructed a custom cultivation chamber illuminated by LED strips that enables us to set the light spectrum and mimic natural sunlight. In our custom cultivation box (Fig. 1A) *Chara* was able to grow continuously, regardless of the season. The light spectrum is shown in Figure 1B and the wiring diagram of LED strips in Figure 1C. In these conditions, *Chara* was maintained for over 5 years. The combination of a custom‐designed, programmable illumination system and a simplified soil/sand medium in our approach ensures consistent growth independent of natural light conditions. This allows for continuous cultivation and experimental reproducibility across seasons. Furthermore, the use of inexpensive, easily available components makes this setup cost‐efficient and feasible for laboratories without specialized infrastructure. These aspects together represent a practical advancement over existing *Chara* species cultivation methods.

The germination protocol presented here is specifically optimized for *C. braunii* strain NIES‐1604. Since *Chara* is widely distributed and grows at various geographical altitudes, its germination requirements may differ from strain to strain (Stross, 1989). In our study, the long dormancy period (6 months) represents the biggest challenge in obtaining enough germinated oospores. In nature, dormancy allows oospores to disperse through water and germinate when conditions are favorable (Casanova & Brock, 1996). The germination rate in our laboratory conditions reached up to 26%, but in nature, it may be higher. As a result, we relied on vegetative propagation to maintain the culture. Additionally, we show that *C. braunii* can germinate on pure agar, without the addition of organic matter, which contradicts previous reports for strain S276 (Holzhausen et al., 2022), further emphasizing strain‐specific requirements.

### Critical Parameters

#### Cultivation box

The critical parameters for successful *C. braunii* cultivation in the illumination box are light intensity, photoperiod, and temperature control. LED strips must be calibrated to deliver ∼30 µmol m^−2^ s^−1^ at the plant surface, as deviations can lead to poor or uneven growth. Maintaining a consistent day and night cycle of 14 hr light and 10 hr dark, with temperatures of 19°C during the dark phase and 22°C during the light phase, is essential for reproducibility. Reliable wiring of the MOSFET modules to the Raspberry Pi ensures stable illumination control, while the inclusion of a fan is necessary to prevent overheating and maintain uniform air circulation within the chamber.

#### Vegetative propagation

For vegetative propagation, strict attention to sterility and medium preparation is crucial. Soil, water, and tools must be sterilized separately to reduce the risk of contamination, while avoiding the formation of a concentrated soil extract that inhibits thallus growth. During handling, thalli should always remain submerged, as even brief drying can impair regeneration. Explants must be positioned carefully, with the basal node buried in the sand to enable rhizoid formation and stable anchorage. Long‐term growth depends on regular culture maintenance, particularly water replacement every 2 to 3 weeks, with increasing volumes as thalli elongate to avoid nutrient depletion and ensure healthy development.

#### Oospore germination

Successful germination of *C. braunii* oospores depends first on the selection of mature reproductive material. Only dark brown to black oospores, harvested from senescing thalli, are competent to germinate. A prolonged stratification period of ∼6 months at 4°C is required to break dormancy; shorter treatments result in poor germination. Because hydrated oospores are fragile, all manipulations must be performed gently to avoid mechanical damage. Disinfection is another critical step. Repeated washes following detergent and bleach treatments are essential to remove residues while still reducing microbial contamination. Finally, although germination can occur on agar medium, development beyond the protonema stage requires transfer to a soil/sand medium, which provides the nutrients necessary for thallus formation.

#### Staining (FM dyes, Calcofluor, Hoechst)

In fluorescent staining protocols, the quality and handling of the biological material are decisive for imaging outcomes. Only freshly harvested, healthy thalli or explants yield reproducible staining, as degraded tissues compromise dye uptake. Concentration and incubation time of FM dyes, Calcofluor White, or Hoechst must be optimized to balance signal strength with background fluorescence; overstaining generates excessive noise, whereas insufficient staining produces weak signals. Careful washing of samples after staining is critical to remove unbound dye and improve image clarity. During mounting for confocal microscopy, special attention must be paid to avoid crushing delicate *Chara* tissues, particularly oospores, by using coverslip spacers or gentle placement. Finally, the choice of developmental stage for staining directly determines the type of information obtained, making precise sample selection an important parameter for successful experiments.

#### Development of generative organs

Antheridia form before oogonia, and their initial development is not visible to the naked eye. In our conditions, antheridia formation began ∼1 week after culture initiation. For early‐stage antheridia observation, examine the apical part of the thallus under a microscope. Mature antheridia enlarge and change color from light green to dark orange. Oogonia forms above antheridia 2 to 3 days later. Mature antheridia release spermatozoids, which swim to the oogonia and fuse with egg cells to form oospores. Fertilization occurs rapidly. For detailed information on antheridia and oogonia formation in *C. braunii*, refer to Sato et al. (2014).

### Troubleshooting

Please see Table 4 for a troubleshooting guide for *Chara* cultivation, germination, and staining.

### Understanding Results

#### Cultivation

**Figure 2:**
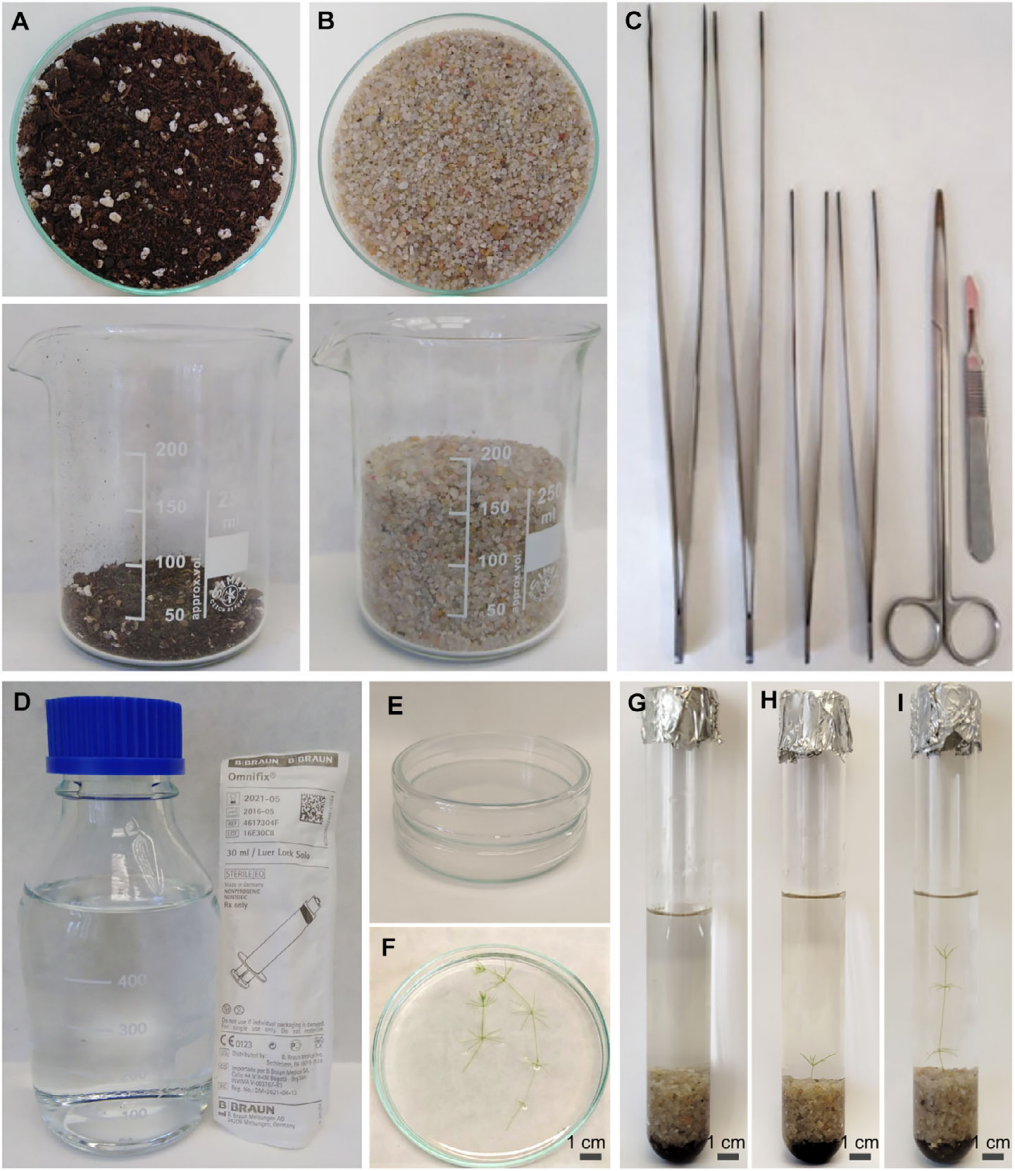
Materials for vegetative propagation of *C. braunii* NIES‐1604. (**A**) Garden soil with the addition of limestone (**B**) 99.9% silica sand. (**C**) Set of metal equipment for propagation. From left to right: 30‐cm laboratory tweezers; 23‐cm laboratory tweezers; Metzenbaum surgical scissors (25‐cm, blunt, straight); metal scalpel with removable blades; (**D**) 500‐ml blue cap bottle and sterile 30‐ml syringe; (**E**) 10‐cm glass Petri dishes; (**F**) *C. braunii* thallus in sterile deionized water used for propagation; (**G**) empty glass tube containing soil/sand medium and 30‐ml of sterile deionized water (*Chara* medium); and (**H**) glass tube containing *Chara* medium and newly propagated thallus segment consisting of 2 nodes connected with an internodal cell. Note that the bottom node is submerged under the sand. (**I**) Glass tube containing *Chara* medium and 2‐week‐old *Chara*. The thallus has elongated and contains 4 nodes with 3 internodal cells in between.

The combination of plain garden soil (Fig. 2A) and silica sand (Fig. 2B) was shown to be suitable for the long‐term growth of *C. braunii*. By adhering to aseptic conditions during propagation using sterile dishes and tools (Figs. 2C‐F), we were able to maintain contamination levels under control, preventing the overgrowth of epiphytes and the collapse of the population. Regular water changes and maintaining a clear medium further reduced contamination to a minimum. In these conditions, growth of propagated *Chara* explant to adult thallus took ∼2 weeks (Figs. 2G‐I). During any experimental design, healthy plants and enough biomass are crucial to obtain. For example, isolation of total protein from *Chara* typically requires ∼1 g of fresh weight biomass, which can only be obtained from multiple thalli grown under consistent and healthy conditions. While material collected from outdoor sources can sometimes be used for experiments, such material is inherently variable and non‐reproducible due to fluctuating environmental conditions. In contrast, our cultivation system maintains stable growth conditions throughout the year, producing homogeneous biomass with minimal variation between growing batches. This reliability enables consistent yields of high‐quality material suitable for molecular and biochemical analyses, which has not been feasible with previous cultivation methods.

#### Germination from oospores

The establishment of a successful germination protocol was a result of germination assays. The first assay was performed on 10 oospores per treatment. However, due to the heterogeneity of the material, this sample size was not large enough to draw definitive conclusions between treatments. Nonetheless, it was evident that a higher concentration of commercial bleach for a shorter time resulted in lower germination (Table 3). To increase the germination percentage, oospores were left to stratify for 6 months in the refrigerator at 4°C. For this experiment (Table 3), the number of oospores per treatment was increased to 30. The prolonged stratification increased the germination rate up to 25 % in one of 2 years of this experiment. Additionally, we show that *C. braunii* NIES‐1604 can germinate on pure agar, without the addition of organic matter, which contradicts previous reports for strain S276 (Holzhausen et al., 2022).

**Table 3:** Test Stratification Length and Disinfection on Germination Efficacy in *C. braunii* Strain NIES‐1604 Oospores

No. spores/No. replications	Stratification	Illumination	Disinfection	Germination % ± *SE* (significance)*^a^*
10/4*^a^*	2 months at 4°C	light	50% bleach; 1 min	8 ± 4 (n.s.)
10/4*^a^*	2 months at 4°C	light	25% bleach; 2 min	8 ± 4 (n.s.)
10/4*^a^*	2 months at 4°C	light	10% bleach; 10 min	8 ± 4 (n.s.)
10/4*^a^*	2 months at 4°C	dark	50% bleach; 1 min	3 ± 3 (n.s.)
10/4*^a^*	2 months at 4°C	dark	25% bleach; 2 min	15 ± 6 (n.s.)
10/4*^a^*	2 months at 4°C	dark	10% bleach; 10 min	23 ± 7 (n.s.)
30/7*^c^*	6 months at 4°C	light	10% bleach; 10 min	17 ± 3 (n.s.)

^*a*^ Germination success was analyzed by GLM with a binomial family of distribution combined with pairwise comparison computed using EMMEANS package in R. n.s.; non‐significant.

^*b*^ Treatments combined with application of auxins [100 nM indole‐3‐acetic acid (IAA), 1‐naphtaleneacetic acid (NAA), and 2,4‐dichlorophenoxyacetic acid (2,‐4D)] with non‐significant effect.

^*c*^ Repeated with independent spore harvests obtained in two different years.

**Table 4:** Troubleshooting Guide for *Chara* Cultivation, Germination, and Staining

Problem	Possible cause	Solution
Uneven or weak growth in cultivation box	Incorrect light intensity or uneven distribution	Calibrate LEDs to ∼30 µmol m⁻² s⁻¹; ensure strips are evenly mounted and functional
Growth inconsistent between replicates	Temperature or photoperiod not precisely maintained	Verify Raspberry Pi programming; confirm chamber temperature remains 19°‐22°C with 14 hr/10 hr ligh/dark cycle
Overheating in cultivation chamber	Insufficient air circulation	Ensure fan is installed and functional; improve airflow within the box
High contamination rates in tube cultures	Incomplete sterilization of soil, tools, or water	Autoclave soil twice, sterilize water and tools separately, and handle under sterile conditions
Cloudy medium inhibiting growth	Excessive soil extract formation	Sterilize soil and water separately; avoid pouring water directly over soil
Explants fail to regenerate	Nodes not in contact with sand or drying during transfer	Bury basal node under sand; keep thalli submerged during handling
Slow or absent oospore germination	Immature or insufficiently stratified oospores	Harvest only dark brown/black oospores; stratify for ∼6 months at 4°C
Oospores damaged during handling	Rough pipetting or manipulation	Use cut pipette tips and gentle handling with fine tweezers
Oospore germination stops at protonema	Growth on agar without nutrients	Transfer to soil–sand medium after germination
Weak or inconsistent staining	Old or unhealthy explants; incorrect dye concentration	Use freshly harvested tissue; adjust dye concentration and incubation time
High background fluorescence	Overstaining or insufficient washing	Reduce dye concentration/incubation; wash thoroughly with sterile water or PBS
Samples collapse under coverslip	Delicate tissues crushed during mounting	Use coverslip spacers for oospores; mount gently with minimal pressure

#### Germinated oospore development

**Figure 3:**
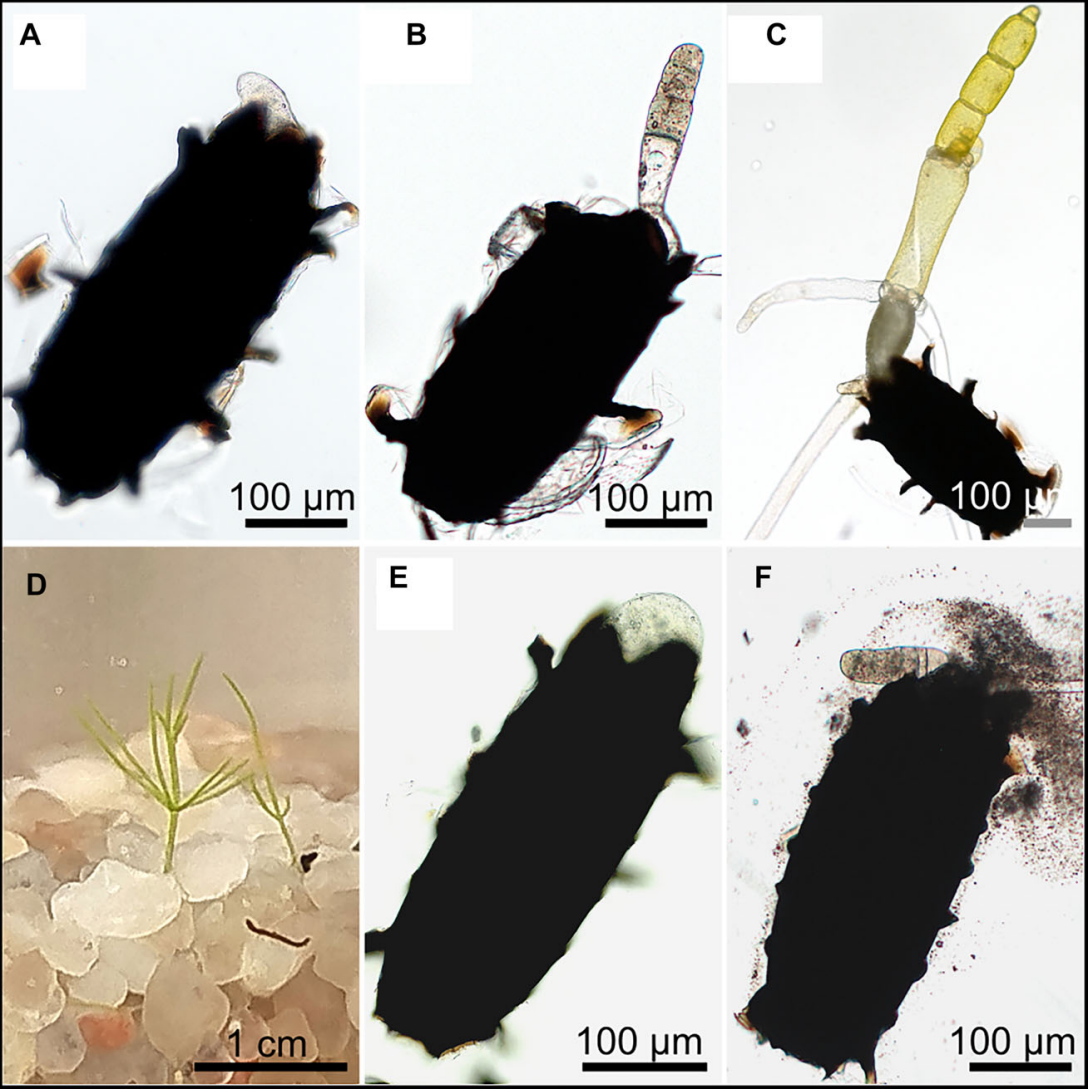
Various stages of germinated oospores. (**A**) Oospore germinated in the agar medium. (**B**) Germinated oospore from agar medium in 6‐cell stage. (**C**) 6‐cell stage germling with elongating cells and the first whorl of branchlets from the first node. (**D**) A proto‐thalli developed from agar‐germinated oospore sown into a soil/sand medium. (**E**) Refrigerator‐germinated stratified oospore. (**F**) A germinated oospore pressed and damaged to release starch granules.

In our experimental conditions, germination began ∼2 weeks after sowing in agar medium. Figure 3A shows the initial protrusion of a 1‐cell rhizoid from the oospore, displaying positive gravitropism, followed by a negatively gravitropic 1‐cell protonema. The transparent protonema undergoes several rounds of cell division (Fig. 3B). The 6‐cell stage germling then begins elongation and forms plastids, becoming photosynthetic (Fig. 3C). The first whorls of branchlets are visible, and secondary rhizoids form at the first node, while the primary rhizoid continues to elongate. At this stage, the protonema can be transferred to soil/sand medium. It is important to place the oospore and rhizoids under the surface of the sand, leaving the green part above the surface.

After another 2 weeks, the second whorl of branchlets develops, and the thallus becomes fully photosynthetic (Fig. 3D). It is important to note that the whole life cycle from germinated oospores is slower than from vegetatively propagated thalli. *Chara* will reach maturity after ∼2 months if grown from germinated oospores, compared to 1 month from propagated thalli. Another key observation is that some oospores in our conditions germinated in the refrigerator after their stratification period had finished (Fig. 3E). However, the oospores could not continue developing due to the low temperature and lack of nutrients. Additionally, when performing microscopy observations of oospores, one must be careful not to apply too much pressure, as germinated oospores are very delicate and easily damaged (Fig. 3F).

#### Fluorescence in vivo staining of plasma membrane, cell wall, and nuclei

**Figure 4:**
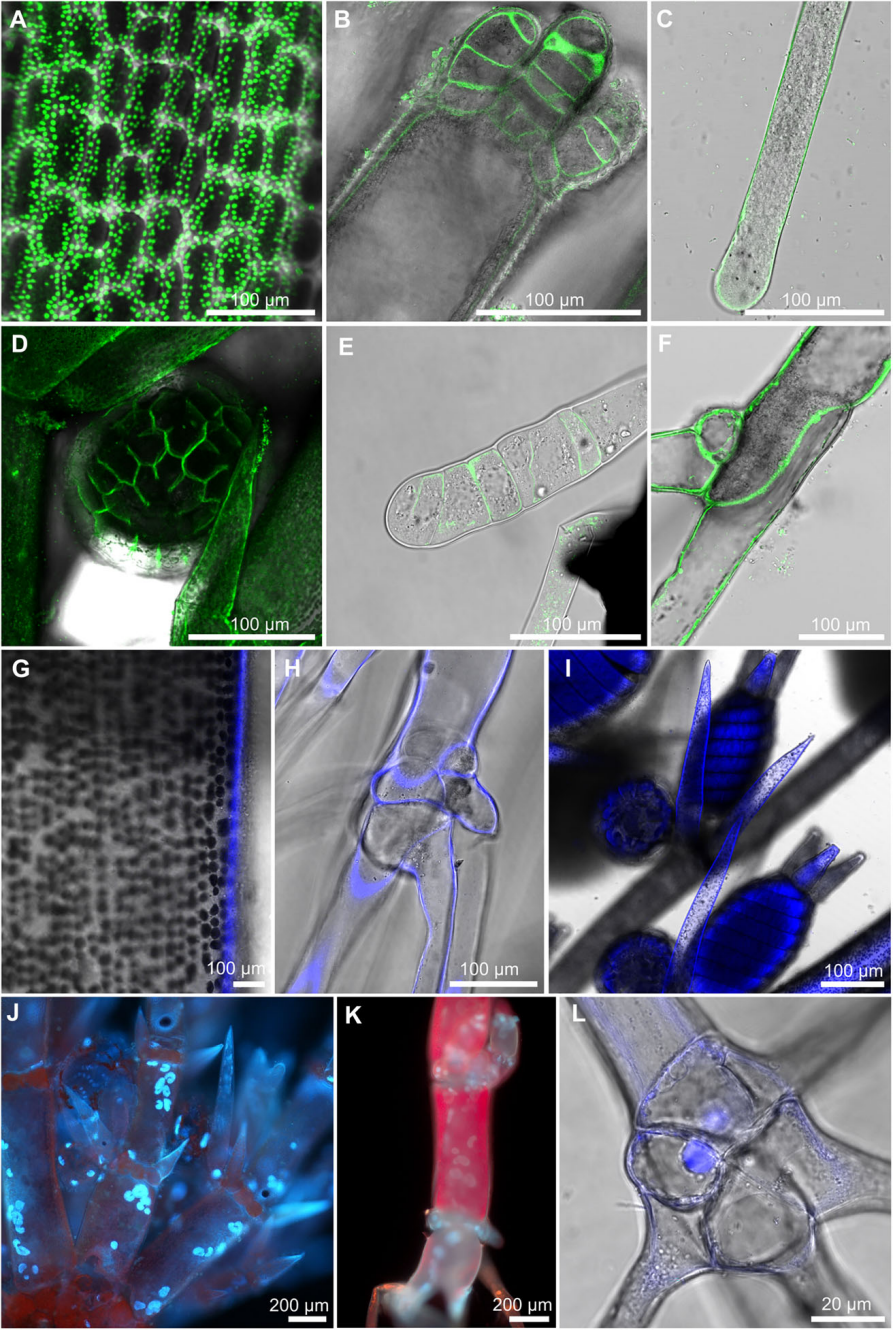
In vivo staining of *C. braunii* cells with fluorescent stains. (**A**‐**F**) In vivo staining of plasma membrane with FM 1‐43 (excitation 488 nm; emission 500‐550 nm). (**A**) Charasome of internodal cell. (**B**) Newly regenerated branchlet. (**C**) Rhizoid tip. (**D**) Antheridium. (**E**) Germinated oospore. (**F**) Branching rhizoid. (**G**‐**I**) Calcofluor White staining of the cell wall (excitation 405 nm; emission 420‐480 nm). (**G**) Internodal cell. (**H**) Branching rhizoid. (**I**) Oogonia and antheridia. (**J**‐**L**) Hoechst staining of the nuclei [(**J‐K**) UV‐excited fluorescence; (**L**) excitation 405 nm, emission 420‐480 nm]. (**J**) Thallus tip. (**K**) Germinated oospore. (**L**) Branching rhizoid.

In *C. braunii*, the plasma membrane forms specialized invaginated structures called charasomes (Fig. 4A). On the Leica SP8 system, FM 1‐43 produced a cleaner signal and higher contrast between the plasma membrane and background fluorescence, whereas FM 4‐64 exhibited stronger chlorophyll autofluorescence in green tissues. Other cell types in *Chara*, such as branches (Fig. 4B) that are formed underground, rhizoids (Fig. 4C and 4F), antheridia (Fig. 4D), and protonema (Fig. 4E), have a “smooth” plasma membrane. Thus, FM 1‐43 is recommended for imaging photosynthetic thalli, while FM 4‐64 remains suitable for rhizoids or early‐stage protonema where chloroplast content is low. Calcofluor White is a suitable dye for cell wall staining in all cell types of *C. braunii*, including internodal cells (Fig. 4G), rhizoids (Fig. 4H), and generative organs (Fig. 4I). It is a non‐toxic alternative to propidium iodide (Flores‐Félix et al., 2015) and can be used in combination with other dyes. Nucleus staining is challenging due to rapid cytoplasmic streaming, and most observations are made on fixed cells or cytoplasmic droplets (Foissner & Wasteneys, 2000). Here, we present an alternative to the fixation method using in vivo Hoechst 33342 dye. This dye easily penetrates the whole thallus of *C. braunii* (Fig. 4J) when incubated in PBS. Germinated oospores contain fewer, larger nuclei (Fig. 4K), while rhizoid nodes contain a single nucleus (Fig. 4L). As internodal cells mature, their nuclei fragment through amitosis resulting in several thousand nuclei per cell (Foissner & Wasteneys, 2000). In contrast, rhizoids do not exhibit multiple nuclei. Additionally, staining of the oospore nucleus was not possible due to the autofluorescence of starch granules and the thick oospore coat.

#### FM 1‐43 provides superior membrane labeling with reduced chloroplast interference compared to FM 4‐64

**Figure 5:**
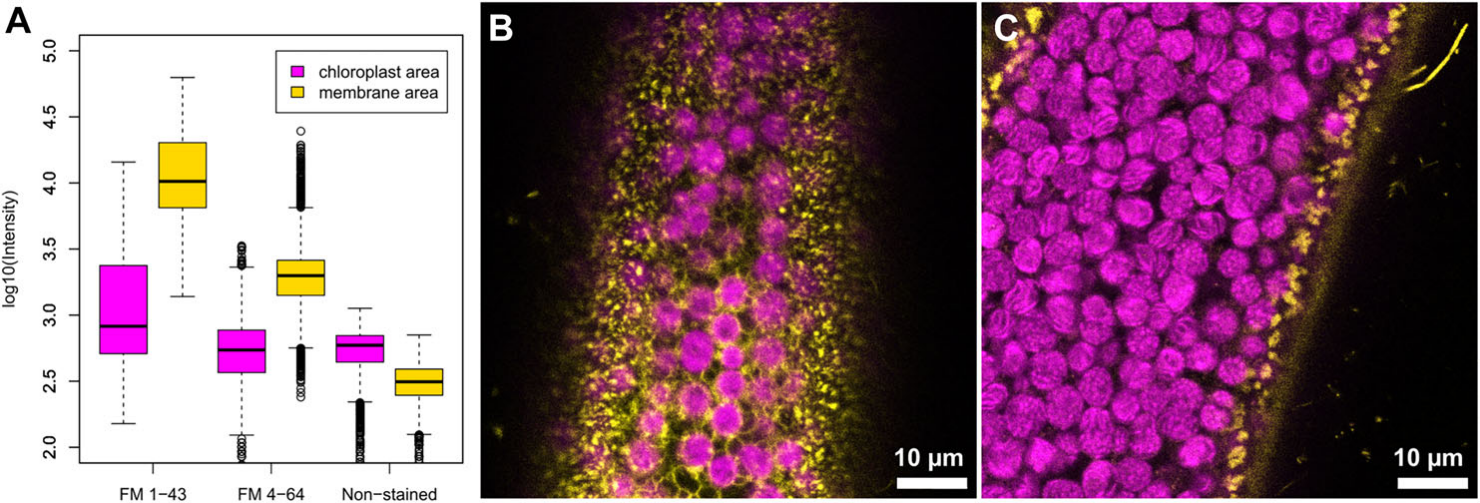
Features of styryl dyes FM 1‐43 and FM 4‐64 in membrane structure staining. (**A**) Signal‐to‐noise comparison for stained and non‐stained cells. Emission intensities in the 570‐620 nm band were measured in FM‐labeled membrane structures (signal, yellow) and in chloroplast regions (noise, magenta). In non‐stained controls, an arbitrarily defined peripheral region of the cell was measured as the “signal”. Representative confocal sections of cells stained with (**B**) FM 1‐43 and with (**C**) FM 4‐64, excited with 488 nm and 561 nm lasers, respectively.

To evaluate the efficiency of styryl dyes in labeling membrane structures in *C. braunii*, we compared the fluorescence patterns and signal‐to‐noise characteristics of FM 1‐43 and FM 4‐64 (Fig. 5). Both dyes effectively stained peripheral membrane regions, but FM 1‐43 yielded a substantially higher membrane‐to‐chloroplast signal ratio than FM 4‐64, indicating superior discrimination between membrane structures and background autofluorescence. Quantitative analysis of emission intensities (log₁₀‐transformed) confirmed this difference, showing higher signal levels in the membrane region and lower background from chloroplasts for FM 1‐43 (Fig. 5A). Representative confocal sections of cells stained with FM 1‐43 (Fig. 5B) and with FM 4‐64 (Fig. 5C) further illustrate that FM 1‐43 provides more distinct membrane labeling with reduced chloroplast interference compared to FM 4‐64. On the Leica SP8 system, FM 1‐43 produced a cleaner signal and higher contrast between the plasma membrane and background fluorescence, whereas FM 4‐64 exhibited stronger chlorophyll autofluorescence in green tissues.

#### Limitations and potential adaptations

While the protocols presented in this article provide a reproducible framework for maintaining and studying *C. braunii* NIES‐1604, several limitations should be acknowledged. The long stratification period (∼6 months) required for efficient oospore germination represents a major temporal bottleneck that may constrain experiments requiring synchronous culture initiation. In addition, germination requirements and culture responses can vary substantially among *Chara* species and even among strains of *C. braunii*, reflecting adaptation to different ecological niches. Therefore, parameters, such as stratification duration, photoperiod, and water chemistry may need to be empirically optimized when applying this system to other *Chara* taxa or related streptophyte algae, e.g., *Nitella* or *Coleochaete*. Nonetheless, the modular design of our cultivation chamber and the use of readily adjustable temperature and light programs provide a flexible platform that can be readily adapted to test species‐specific growth requirements and comparative developmental studies across streptophytes.

### Time Considerations

The whole life cycle of *Chara*, from germination of oospores to full maturation of adult plants can last ∼3 months, without the dormancy included. The stratification period of oospores is the critical issue. However, with the proper planning and ensuring that mature oospores are harvested regularly and placed for stratification, the continuous growth of *Chara* should not be an issue.

For vegetative propagation, the preparation of the medium and sterilization of laboratory materials can be initiated 1 day prior, while propagation in the sterile box is typically completed between 1 and 3 hr, depending on the number of samples needed to be propagated.

The oospore disinfection and sowing take up to 1 hr. First oospores are washed with Tween 20 which takes 15 min, then 8 min of disinfection and another 5 to 10 min of washing from bleach. If oospores are used immediately, the sowing takes 30 to 60 min.

### Author Contributions

**Katarina Kurtović**: Conceptualization; investigation; methodology; writing—original draft; writing—review and editing. **Jan Petrášek**: Conceptualization; supervision; writing—review and editing. **Stanislav Vosolsobě**: Conceptualization; methodology; resources; writing—original draft.

### Conflict of Interest

The authors declare no competing interests.

## Data Availability

The Python code for the illumination unit is available from the repository at: https://github.com/vosolsob/illumination. Fluorescence intensity was measured using the ImageJ macro Intensity_.ijm available at: https://github.com/vosolsob/ImageJ_macros/.

## References

[cpz170279-bib-0001] Absolonova, M. , Foissner, I. , & Sommer, A. (2019). Cubic plasma membrane domains stabilize and restrict zones for pH band formation in Chara internodal cells. Botany Letters, 166(3), 283–293. 10.1080/23818107.2018.1544508

[cpz170279-bib-0002] Beilby, M. J. , Bisson, M. A. , & Schneider, S. C. (2022). How Characean algae take up needed and excrete unwanted ions – An overview explaining how insights from electrophysiology are useful to understand the ecology of aquatic macrophytes. Aquatic Botany, 181, 103542. 10.1016/j.aquabot.2022.103542

[cpz170279-bib-0003] Bowman, J. L. (2022). The origin of a land flora. Nature Plants, 8(12), 1352–1369. 10.1038/s41477-022-01283-y 36550365

[cpz170279-bib-0004] Casanova, M. T. , & Brock, M. A. (1996). Can oospore germination patterns explain charophyte distribution in permanent and temporary wetlands? Aquatic Botany, 54(4), 297–312. 10.1016/0304-3770(96)01032-7

[cpz170279-bib-0005] Chau, R. , Bisson, M. A. , Siegel, A. , Elkin, G. , Klim, P. , & Straubinger, R. M. (1994). Distribution of charasomes in Chara: Re‐establishment and loss in darkness and correlation with banding and inorganic carbon uptake. Functional Plant Biology, 21(1), 113–123. 10.1071/pp9940113

[cpz170279-bib-0006] Current Protocols . (2006). Commonly Used Reagents. Current Protocols in Microbiology, 00, A.2A.1–A.2A.15. 10.1002/9780471729259.mca02as00

[cpz170279-bib-0007] Delwiche, C. F. , & Cooper, E. D. (2015). The evolutionary origin of a terrestrial flora. Current Biology, 25(19), R899–R910. 10.1016/j.cub.2015.08.029 26439353

[cpz170279-bib-0008] Domozych, D. S. , Popper, Z. A. , & Sørensen, I. (2016). Charophytes: Evolutionary Giants and Emerging Model Organisms. Frontiers in plant science, 7, 1470. 10.3389/fpls.2016.01470 27777578 PMC5056234

[cpz170279-bib-0009] Domozych, D. S. , & Bagdan, K. (2022). The cell biology of charophytes: Exploring the past and models for the future. Plant Physiology, 190(3), 1588–1608. 10.1093/plphys/kiac390 35993883 PMC9614468

[cpz170279-bib-0010] Flores‐Félix, J. D. , Menéndez, E. , Marcos‐García, M. , Celador‐Lera, L. , & Rivas, R. (2015). Calcofluor white, an alternative to propidium iodide for plant tissues staining in studies of root colonization by fluorescent‐tagged Rhizobia. Journal of Advances in Biology & Biotechnology, 65–70. 10.9734/JABB/2015/12444

[cpz170279-bib-0011] Foissner, I. , & Wasteneys, G. (2000). Nuclear crystals, lampbrush‐chromosome‐like structures, and perinuclear cytoskeletal elements associated with nuclear fragmentation in characean internodal cells. Protoplasma, 212, 146–161. 10.1007/BF01282916

[cpz170279-bib-0012] Forsberg, C. (1965). Sterile germination of oospores of *Chara* and seeds of *Najas marina* . Physiologia Plantarum, 18(1), 128–137. 10.1111/j.1399-3054.1965.tb06875.x

[cpz170279-bib-0013] Heß, D. , Heise, C. M. , Schubert, H. , Hess, W. R. , & Hagemann, M. (2023). The impact of salt stress on the physiology and the transcriptome of the model streptophyte green alga *Chara braunii* . Physiologia Plantarum, 175(6), e14123. 10.1111/ppl.14123 38148211

[cpz170279-bib-0014] Heß, D. , Holzhausen, A. , & Hess, W. R. (2023). Insight into the nodal cells transcriptome of the streptophyte green alga *Chara braunii* S276. Physiologia Plantarum, 175(5), e14025. 10.1111/ppl.14025 37882314

[cpz170279-bib-0015] Holzhausen, A. , Stingl, N. , Rieth, S. , Kühn, C. , Schubert, H. , & Rensing, S. A. (2022). Establishment and optimization of a new model organism to study early land plant evolution: Germination, cultivation and oospore variation of *Chara braunii* Gmelin, 1826. Frontiers in Plant Science, 13, 987741. 10.3389/fpls.2022.987741 36438147 PMC9691404

[cpz170279-bib-0016] Klima, A. , & Foissner, I. (2008). FM Dyes Label Sterol‐Rich Plasma Membrane Domains and are Internalized Independently of the Cytoskeleton in Characean Internodal Cells. Plant and Cell Physiology, 49(10), 1508–1521. 10.1093/pcp/pcn122 18757863

[cpz170279-bib-0017] Kurtović, K. , Schmidt, V. , Nehasilová, M. , Vosolsobě, S. , & Petrášek, J. (2024). Rediscovering Chara as a model organism for molecular and evo‐devo studies. Protoplasma, 261(2), 183–196. 10.1007/s00709-023-01900-3 37880545

[cpz170279-bib-0018] Nishiyama, T. , Sakayama, H. , de Vries, J. , Buschmann, H. , Saint‐Marcoux, D. , Ullrich, K. K. , Haas, F. B. , Vanderstraeten, L. , Becker, D. , Lang, D. , Vosolsobě, S. , Rombauts, S. , Wilhelmsson, P. K. I. , Janitza, P. , Kern, R. , Heyl, A. , Rümpler, F. , Villalobos, L. I. A. C. , Clay, J. M. , … Rensing, S. A. (2018). The *Chara* genome: Secondary complexity and implications for plant terrestrialization. Cell, 174(2), 448–464. 10.1016/j.cell.2018.06.033 30007417

[cpz170279-bib-0019] Sabbatini, M. R. , Argüello, J. A. , Fernández, O. A. , & Bottini, R. A. (1987). Dormancy and growth‐inhibitor levels in oospores of *Chara contraria* A. Braun ex Kütz. (Charophyta). Aquatic Botany, 28(2), 189–194. 10.1016/0304-3770(87)90040-4

[cpz170279-bib-0020] Sato, M. , Sakayama, H. , Sato, M. , Ito, M. , & Sekimoto, H. (2014). Characterization of sexual reproductive processes in *Chara braunii* (Charales, Charophyceae). Phycological Research, 62(3), 214–221. 10.1111/pre.12056

[cpz170279-bib-0021] Schmölzer, P. M. , Höftberger, M. , & Foissner, I. (2011). Plasma membrane domains participate in pH banding of *Chara* internodal cells. Plant & Cell Physiology, 52(8), 1274–1288. 10.1093/pcp/pcr074 21659328 PMC3153728

[cpz170279-bib-0022] Stross, R. G. (1989). The temporal window of germination in oospores of *Chara* (Charophyceae) following primary dormancy in the laboratory. New Phytologist, 113(4), 491–495. 10.1111/j.1469-8137.1989.tb00360.x

